# Evolutionary Analyses of Gene Expression Divergence in *Panicum hallii*: Exploring Constitutive and Plastic Responses Using Reciprocal Transplants

**DOI:** 10.1093/molbev/msad210

**Published:** 2023-09-20

**Authors:** Govinal Badiger Bhaskara, Taslima Haque, Jason E Bonnette, Joseph D Napier, Diane Bauer, Jeremy Schmutz, Thomas E Juenger

**Affiliations:** Department of Integrative Biology, The University of Texas at Austin, Austin, TX, USA; Department of Integrative Biology, The University of Texas at Austin, Austin, TX, USA; Department of Integrative Biology, The University of Texas at Austin, Austin, TX, USA; Department of Integrative Biology, The University of Texas at Austin, Austin, TX, USA; DOE Joint Genome Institute, Lawrence Berkeley National Laboratory, Berkeley, CA, USA; DOE Joint Genome Institute, Lawrence Berkeley National Laboratory, Berkeley, CA, USA; Genome Sequencing Center, HudsonAlpha Institute for Biotechnology, Huntsville, AL, USA; Department of Integrative Biology, The University of Texas at Austin, Austin, TX, USA

**Keywords:** local adaptation, expression divergence, common garden, 3′ TagSeq, *Q*_PC_

## Abstract

The evolution of gene expression is thought to be an important mechanism of local adaptation and ecological speciation. Gene expression divergence occurs through the evolution of cis- polymorphisms and through more widespread effects driven by *trans*-regulatory factors. Here, we explore expression and sequence divergence in a large sample of *Panicum hallii* accessions encompassing the species range using a reciprocal transplantation experiment. We observed widespread genotype and transplant site drivers of expression divergence, with a limited number of genes exhibiting genotype-by-site interactions. We used a modified *F*_ST_–*Q*_ST_ outlier approach (*Q*_PC_ analysis) to detect local adaptation. We identified 514 genes with constitutive expression divergence above and beyond the levels expected under neutral processes. However, no plastic expression responses met our multiple testing correction as *Q*_PC_ outliers. Constitutive *Q*_PC_ outlier genes were involved in a number of developmental processes and responses to abiotic environments. Leveraging earlier expression quantitative trait loci results, we found a strong enrichment of expression divergence, including for *Q*_PC_ outliers, in genes previously identified with cis and cis–environment interactions but found no patterns related to *trans*-factors. Population genetic analyses detected elevated sequence divergence of promoters and coding sequence of constitutive expression outliers but little evidence for positive selection on these proteins. Our results are consistent with a hypothesis of cis-regulatory divergence as a primary driver of expression divergence in *P. hallii*.

## Introduction

Gene expression variation plays a major role in phenotypic evolution, both within and between species, facilitating adaptation to their native habitats (Fay and Wittkopp 2008; Gilad et al. 2006; Necsulea and Kaessmann 2014; Nourmohammad et al. 2017; Price et al. 2022; Romero et al. 2012; Whitehead and Crawford 2006a, 2006b). Gene expression is a dynamic trait that responds to environmental changes, leading to plasticity in morphological, physiological, and fitness traits. As populations adapt to changing environments, they can exhibit constitutive or plastic expression divergence. Constitutive expression divergence may occur when plasticity is costly or maladaptive, whereas plasticity can be favored when the environment is heterogeneous and requires multiple optimal responses throughout an organism's life cycle or when environmental cues are predictable (Hendry 2015; Murren et al. 2015; Mäkinen et al. 2017; Li et al. 2019). When the environmental response is heritable and plasticity is beneficial, natural selection can reinforce it leading to evolution of plastic expression (Crispo 2007; Campbell-Staton et al. 2021). This adaptive expression plasticity can play a major role in enhancing resilience to climate change (Nicotra et al. 2010; Brooker et al. 2022). However, there is currently a lack of empirical research that investigates adaptive constitutive and plastic expression divergence in natural populations and their underlying regulatory architectures.

Both *cis*- and *trans*-acting regulatory architectures contribute significantly to expression variation (Fay and Wittkopp 2008). Cis-regulatory elements (CREs) are modular and allow expression changes specific to tissues, life stages, or environmental conditions, whereas *trans*-acting regulatory mutations influence gene regulatory networks with wider connectivity and tend to have larger pleiotropic effects (Prud'homme et al. 2007; Metzger et al. 2017; Vande Zande et al. 2022). This has led to the hypothesis that cis-variants can accumulate over time since they exhibit less deleterious pleiotropy as compared with *trans*-variants (Prud'homme et al. 2007). However, empirical studies suggest that the relative contributions of *cis*- and *trans*-variants for shorter (within species) and longer evolutionary scale (between species) vary considerably across different model systems (Stern and Orgogozo 2008). For instance, in yeast, flies and Arabidopsis, cis-variants contribute more to interspecies differences in gene expression than *trans*-variants (Ronald et al. 2005; Wittkopp et al. 2008; Tirosh et al. 2009; Shi et al. 2012), whereas in maize, Mimulus, and *Panicum hallii* (a perennial grass), cis-variants account for a greater proportion of intraspecies gene expression variation (Schadt et al. 2003; Guo et al. 2004; Springer and Stupar 2007; Lovell et al. 2016; Lovell et al. 2018; Brown and Kelly 2021). This variability may be attributed to factors such as genetic diversity, selective pressure, divergence time, environmental heterogeneity, and even the methods used to estimate expression variation (Li and Burmeister 2005; Meiklejohn et al. 2014). Nevertheless, given the pleiotropic constraints, it is suggested that purifying selection may strongly shape *trans*-variants, whereas positive selection contributes significantly to cis expression variation leading to adaptive divergence (Schaefke et al. 2013).

Divergence in gene expression has been suggested to correlate with the molecular evolution of proteins (Ingvarsson 2007; Slotte et al. 2011). However, the relationship has been the subject of much debate due to contrasting results between studies. For example, Renaut et al. (2012) and Moyers and Rieseberg (2013) found no significant relationship between these two factors. Additionally, the types or direction of selection driving this association also varies between studies (Hodgins et al. 2016). In Drosophila, positive selection has been proposed as a factor driving the association between expression divergence and sequence evolution, whereas in mammals and pines, the weak association between expression divergence and sequence evolution has been attributed to relaxed purifying selection and genetic drift (Nuzhdin et al. 2004; Liao and Zhang 2006; Hodgins et al. 2016).The reasons for this variability remain unclear, but differences in sequence evolution, the efficacy of selection, and the timescale of evolution are likely contributors to the observed patterns.

Perennial grasses provide a unique opportunity to study local adaptation. *Panicum hallii*, a C4 perennial bunchgrass, serves as an emerging model system for investigating local adaptation due to its diverse ecological habitats. The habitat range of this grass varies greatly by precipitation and soil salinity gradients (Lowry et al. 2013; Palacio-Mejía et al. 2021; Haque et al. 2022). The species comprises two major ecotypes that are morphologically divergent: coastal ecotypes are typically confined along the Gulf of Mexico with higher soil moisture and salinity, whereas inland ecotypes are found across the drier xeric habitats of the south–central and western United States (Lowry et al. 2013). Gene expression changes have been implicated in adaptation to abiotic stresses such as drought and salinity using representative inland and coastal ecotypes of *P. hallii* (Lovell et al. 2016; Lovell et al. 2018; Haque et al. 2022). For instance, the expression of drought-responsive genes between inland and coastal ecotypes of *P. hallii* was influenced by CREs (Lovell et al. 2016). Moreover, over 50% of transcripts in *P. hallii* exhibited cis expression quantitative loci (eQTL), indicating the prevalence of cis expression divergence between ecotypes (Lovell et al. 2018). However, the molecular evolution of constitutive and plastic expression divergence across different native environments within this species as well as many other plant model systems remains unexplored.

In this study, we conducted a field reciprocal transplant experiment using natural genotypes of *P. hallii*. Our objectives were to i) detect gene expression variation resulting from genetic differences or plastic responses to the environment, along with their interactions, ii) dissect the heritable constitutive and plastic components of gene expression variation using an outlier-based testing framework (Josephs et al. 2019) designed to detect divergent selection on gene expression, and iii) evaluate the relationship between constitutive gene expression divergence and the rate of sequence and protein evolution. We identified hundreds of genes with constitutive expression divergence beyond neutral expectation, but we detected a very weak signal for adaptive plastic divergence. Moreover, our results suggest that the genes exhibiting constitutive expression divergence in our study were not under positive selection but evolve faster compared with other background genes due to relaxed purifying selection.

## Results

### Population Genetic Analysis of *P. hallii*

**Fig. 1.**
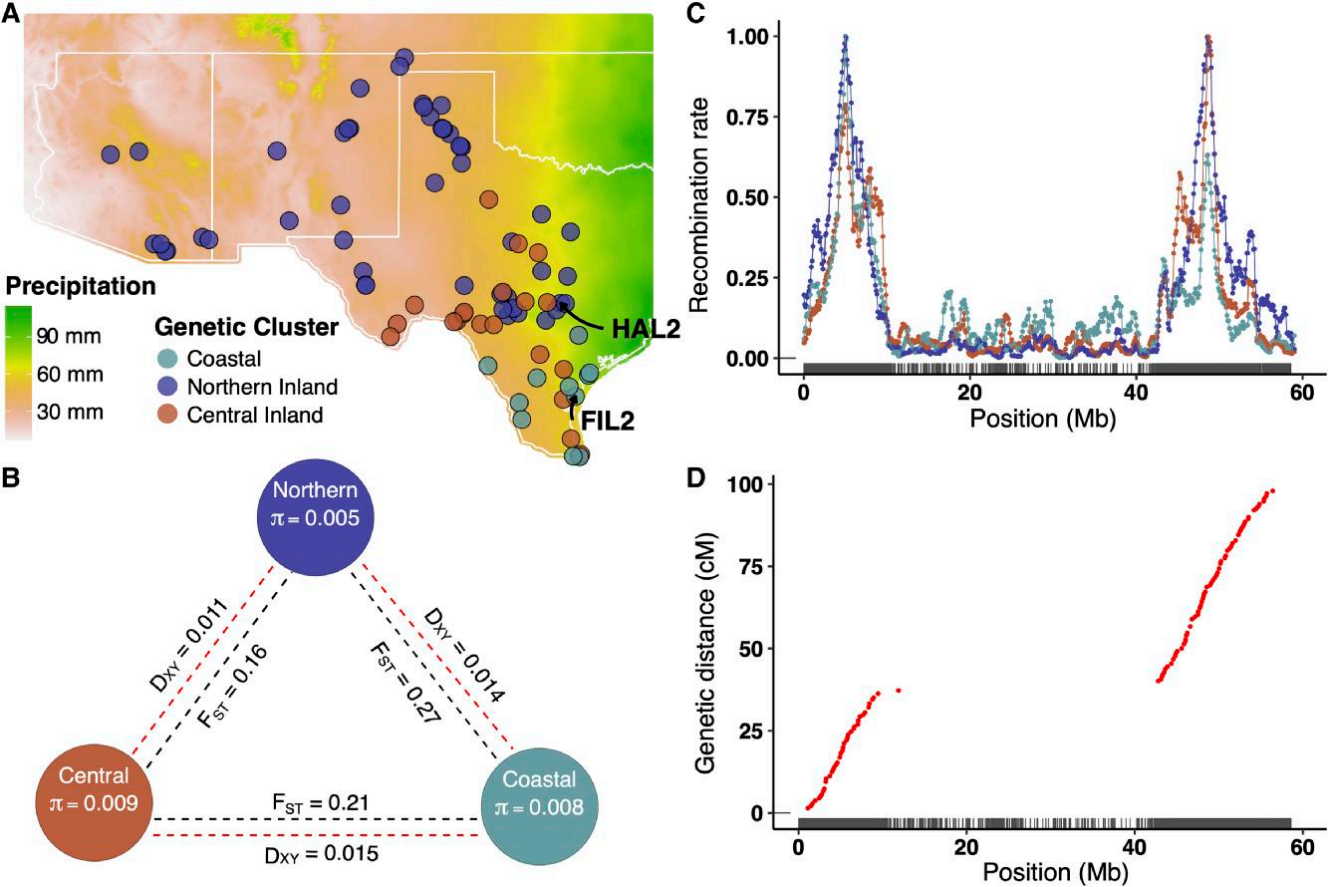
Genetic analysis identified three major genetic clusters of *P. hallii*. (*A*) Genetic structure analysis and geographical distribution of *P. hallii* populations. The background Raster plot was constructed from the average annual precipitation (BIO12) of 2.5 min spatial resolution (http://worldclim.org/version2). Points represent the origin of collections for each accession. Point colors represent genetic subpopulations. Two major genetic clusters (Coastal and Inland) were detected by DAPC of 86 accessions of *P. hallii*. Subsequent Bayesian hierarchical cluster analysis (STRUCTURE) inferred two genetic clusters within the inland population (Northern Inland, dark blue; Central Inland, brick red). Coastal populations (light blue) were plotted based on the origin of collection site without ancestral population inference. (*B*) Genome-wide nucleotide diversity within genetic cluster (π) and divergence statistics (*F*_ST_, *D*_XY_) between genetic clusters. (*C*) Recombination landscape of individual genetic cluster across chromosomes (chromosome 1 as an example). Population-scaled recombination rate was plotted on the *y*-axis, and the *x*-axis represents the physical position of the chromosome. The rug at the bottom of the figure represents gene density. (*D*) Genetic map of *P. hallii* from an RIL population (chromosome 1 as an example). The *x*-axis represents the physical distance, and the *y*-axis represents genetic distance. The gap between the arms of chromosome 1 depicts the heterochromatin region which exhibits low recombination rate (panel *C*).

Natural populations of *P. hallii* exhibit substantial divergence in their morphology and physiology (Lowry et al. 2013) which could be due to historical demographic process and/or adaptation to ecologically different native habitats. To study the role of gene expression divergence in adaptive differentiation, we performed a field reciprocal transplant experiment on 86 genotypes sampled from the natural range of coastal and inland habitats (supplementary table S1, Supplementary Material online). Discriminant analysis of principal components (DAPC) on a random set of 50,000 putatively neutral single nucleotide polymorphisms (SNPs) from the sampled population inferred two major genetic clusters that were consistent with two previously described ecotypes (var. *hallii* and var. *filipes*) (fig. 1*A*, Lovell et al. 2018). Var. *hallii* is distributed over a much wider inland geographic area from Texas to Arizona (hereafter referred to as inland cluster; fig. 1*B*, dark blue and red points), whereas *var. filipes* is restricted to the southeastern portion of Texas (hereafter referred to as the coastal cluster; fig. 1*B*, light blue points). Given the small sample size of coastal cluster (*n* = 11) from a relatively limited geographic area, we chose to focus subsequent hierarchical cluster analysis on the inland population only. Bayesian clustering analysis implemented in STRUCTURE supports two subclusters of the inland collections (*K* = 2) according to the Δk method (supplementary fig. S1 and table S1, Supplementary Material online). We designated these two clusters as the “Northern” (*n* = 53) and “Central” (*n* = 22) clusters. The Northern cluster occurs from Central Texas to Arizona spanning a steep precipitation gradient (fig. 1*A*, dark blue points), whereas the Central cluster is primarily located in Central Texas (fig. 1*A*, dark red points). These three genetic clusters were congruent with the major clusters found in earlier studies of *P. hallii* natural accessions (Lovell et al. 2018; Palacio-Mejía et al. 2021). However, Lovell et al. (2018) and Palacio-Mejía et al. (2021) found finer groups within each cluster likely as a result of their denser sampling. Lovell et al. (2018) estimated the divergence time of the inland and coastal populations of *P. hallii* as over a million year. In order to polarize the ancestry between inland and coastal genetic clusters, we constructed a phylogenetic tree from the concatenated alignment of the one-to-one orthologs among one inland and one coastal *P. hallii* genome reference along with *Panicum virgatum* and *Setaria viridis*. The ancestral state was inferred using *P. virgatum* and *Setaria* as outgroups. We reasoned that the more ancestral ecotype would contain a greater proportion of ancestral allele states and use this framework to test for the order of divergence. However, we found equivocal results as the proportion of ancestral alleles to derived alleles was roughly equal for both inland and coastal populations (95% bootstrapped confidence interval [CI] for the probability that coastal genetic cluster is ancestral = 0.47–0.49).

To investigate genetic differentiation within *P. hallii*, we estimated relative (*F*_ST_) and an absolute measure of divergence (*D*_XY_) between genetic clusters and nucleotide diversity (π) within each cluster at 20 Kb nonoverlapping windows genome-wide. Relative diversity varied significantly (*P* < 0.05) among all three possible pairwise contrasts of genetic clusters (fig. 1*B*). The highest *F*_ST_ was observed in Coastal–Northern contrast (mean *F*_ST_ = 0.27), followed by the Coastal–Central contrast (*F*_ST_ = 0.21), with the least relative divergence observed in the Central–Northern comparisons (*F*_ST_ = 0.16). Similar to relative diversity, the absolute measure of divergence among genetic clusters was significantly different for all three pairwise comparisons (*P* < 0.05). However, here, the Coastal–Central contrast revealed the greatest divergence (mean *D*_XY_ = 0.015), followed by the Coastal–Northern contrast (mean *D*_XY_ = 0.014), and finally the Central–Northern contrast (mean *D*_XY_ = 0.011). The highest nucleotide diversity was observed in the Central cluster (mean π = 0.009), followed by Coastal (mean π = 0.008), and Northern clusters (mean π = 0.005). Overall, we observed Coastal versus Inland pairs were more diverged compared with inland clusters and the Northern cluster had relatively low nucleotide diversity than the other genetic clusters.

Recombination rate has been reported to vary between populations and along chromosomes in many plant species (Dreissig et al. 2019; Schreiber et al. 2022), and can be a major driver of molecular evolution (Dapper and Payseur 2017). In particular, regions of low recombination are often associated with reduced genetic diversity and reduced effectiveness of natural selection. We estimated the mean scaled recombination rate (ρ) for each genetic cluster based on population genetic analyses of whole genome resequencing data. We found significant difference in ρ between genetic clusters of *P. hallii* (post hoc adjusted *P* < 0.05): the highest ρ was estimated for Northern genetic cluster (ρ = 0.33/kb) followed by Central (ρ = 0.24/kb) and Coastal genetic cluster (ρ = 0.2/kb). Noticeably, we observed an elevated recombination rate around euchromatin regions (near the distal end of the chromosome) compared with heterochromatin regions near the pericentromeric regions (Pearson's correlation coefficient of population-scaled recombination rate and gene density for Coastal, Central, and Northern genetic cluster were 0.4, 0.5, and 0.7, respectively; *P* < 2.2e^−16^) (fig. 1*C*; supplementary fig. S2, Supplementary Material online). This landscape of recombination was congruent with the number of crossover events estimated from a recombinant inbred line (RIL) population (HAL2 × FIL2; Khasanova et al. 2019) generated from Northern and Coastal reference parents (Spearman's rank correlation coefficient = 0.5; *P* < 2.2e^−16^) (fig. 1*D*). The highly recombinant region encompassed almost half (46%) of the genome of *P. hallii* (supplementary table S2, Supplementary Material online) and contains the vast majority of coding genes (82%; 27,271). Nevertheless, 5,992 genes were found in the largely pericentromeric regions.

### Plant Water Status and Transcriptome Changes in Response to Genetic Variation and Transplant Sites

**Fig. 2.**
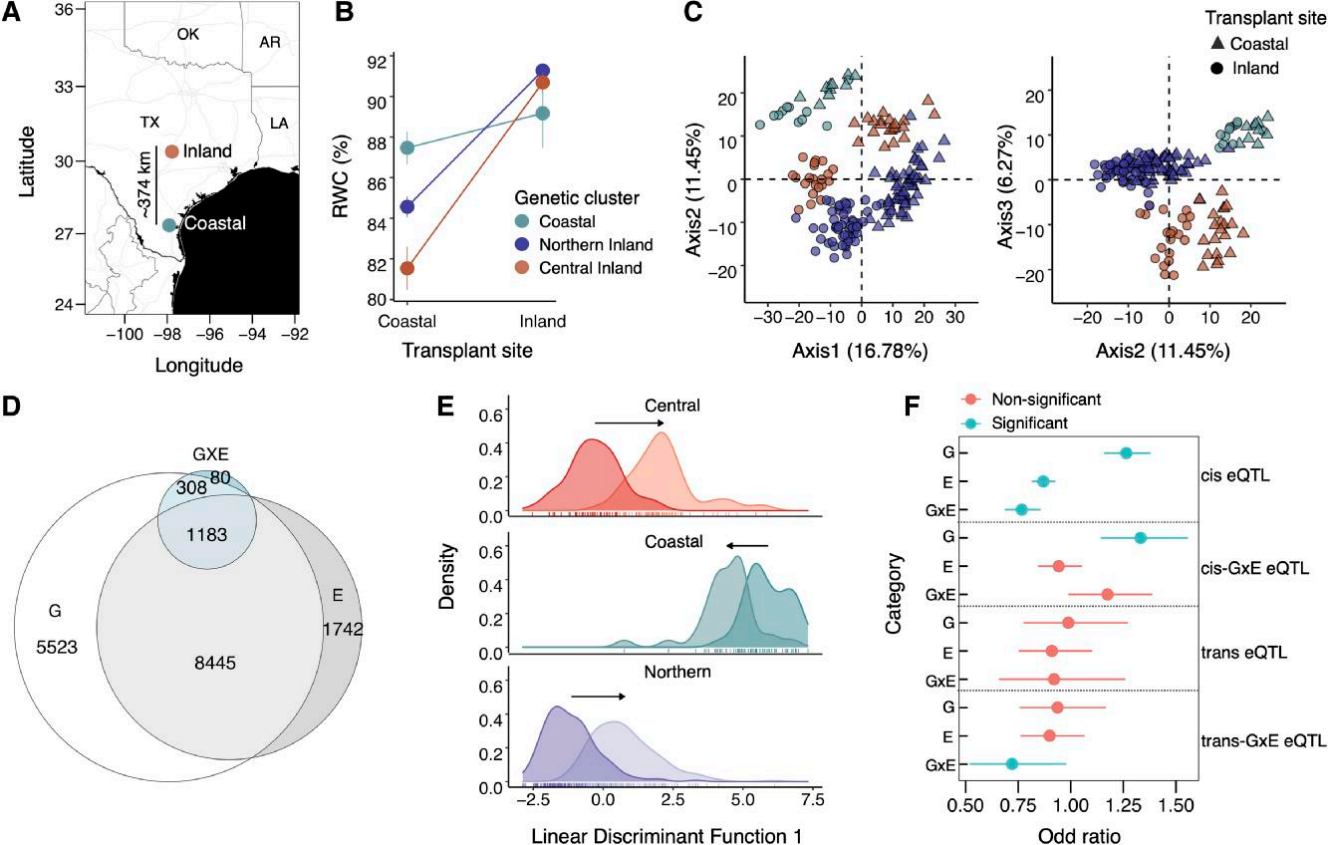
Effects of population and transplant site on gene expression. (*A*) Map of study sites at the Central (Inland) and South Texas (Coastal) locations. (*B*) Reaction norm plots of RWC among populations in response to transplant sites: the *x*- and *y*-axes represent transplant sites and mean RWC for a given genetic cluster, respectively. Points are colored by the genetic clusters. The vertical line centered at the mean indicates standard error (SE). (*C*) PCA of gene expression variation for the top 1,000 most variable genes. Each point represents an accession at the transplant site. The shape of a point represents the transplant site, and the color represents genetic clusters as shown in *B*. (*D*) Venn diagrams showing gene expression distribution for genotype (G), field environment (E), and genotype-by-environment interactions (G×E) for given population contrasts. (*E*) DAPC on total transcriptomic space representing the effect of origin/genetic cluster on expression plasticity. The *x*-axis represents the linear discriminant function in multivariate space along which the difference between the native habitat and transplant site is maximized. Each panel depicts the distributions of DAPC score of focal genetic cluster at the native site (darker shade) and at the transplant site (lighter shade). The distance between the mean of a focal genetic cluster at home to the mean at the transplant site was marked by arrows which represents transcriptomic plasticity. Plasticity was significantly different for all three pairwise comparisons (adjusted *P* value < 0.05). (*F*) Enrichment of DEGs with different categories of expression QTL (eQTL) detected by Lovell et al. (2018). The odd ratio was estimated using Fisher's exact test for the presence of eQTL in a given factor (G, E, or G×E) compared with the expressed background genes.

We used a field reciprocal transplant experiment to study the physiology and gene expression variability among different genetic clusters grown in native and foreign habitats of origin (fig. 2*A*). We assessed plant water status by measuring leaf relative water content (RWC) at the time of tissue harvest for transcriptome sampling. RWC indicates plant water status in terms of the hydration state of plant, and it can be a strong driver of plant performance and gene expression changes (Jones 2007 ; Des Marais et al. 2013). We analyzed RWC for the effect of genetic clusters (G), field environment (E), and their interaction (G×E). In this framework, we interpret a significant main effect of G as constitutive divergence in RWC, a significant effect of E as plastic responses of RWC to planting location, and a significant interaction as genetic variation in the plastic response of genotypes to planting sites in terms of water status. We observed a significant G (*F*_(df = 2)_ = 4.032, *P<* 0.05), E (*F*_(df = 1)_ = 147.3, *P* < 0.001), and G×E (*F*_(df = 2)_ = 6.9, *P <* 0.01) for RWC (fig. 2*B*) revealing genetic variation in whole plant physiology in response to our transplant locations. Overall, the three genetic clusters diverge in RWC at the coastal site (*F*_(df = 2)_ = 8.184, *P<* 0.001) and differ little at the inland site (*F*_(df = 2)_ = 0.7, *P>* 0.1). At the coastal site, the native coastal cluster maintained the highest RWC, followed by Northern and Central clusters.

Similar to plant water status, transcriptomic changes can be responsive to environmental shifts due to transplantation to foreign habitat. The global patterns of expression variation based on a principal component analysis (PCA) detected clear expression differentiation among field environments and *P. hallii* populations (fig. 2*C*). PCA axis 1 (explaining 16.78% variance) was associated with a strong environment effect, whereas axes 2 (11.45% variance) and 3 (6.27% variance) were related to population divergence in gene expression. Overall, variation in gene expression was in concordance with the three distinct genetic clusters of *P. hallii* detected from our population genetic analysis. As such, we first tested for the global effect of G, E, and G×E on each expressed gene by factorial model which includes all three genetic clusters across two field environments. We found a strong transcriptomic response of genetic cluster (15,656 genes with significant G effect) and plasticity (12,387 genes with significant E effect), with many fewer genes exhibiting genetic cluster–dependent plastic response across transplant sites (1,877 genes with significant G×E effect) (fig. 2*D*; supplementary table S3, Supplementary Material online). Similarly, G×E explained a much lower proportion of variance in gene expression than G or E (fig. 2*D*; supplementary fig. S3*A*, Supplementary Material online). Noticeably, we observed enrichment of G category genes and depletion of G×E category genes at low-recombining regions of the genome compared with the highly recombining regions (*P* < 0.05). However, no significant enrichment was found for plastic genes (E).

Subsequently, we conducted post hoc pairwise comparisons between genetic clusters and transplant sites on each expressed gene. Consistent with the global analysis, pairwise analysis that includes two genetic clusters at a time also showed strong G and E transcriptomic responses, and only a few hundred genes exhibited significant G×E effects (supplementary tables S4, S5, and S6, Supplementary Material online). Similarly, G and E explained a relatively higher proportion of variance in gene expression than G×E for all three contrasts (supplementary fig. S3*B*, Supplementary Material online). In addition, we also asked which genes responded similarly across all genetic clusters to the transplant site. We detected 2,950 genes (supplementary table S7, Supplementary Material online) with consistent plastic effects to the transplant site across each genetic clusters indicating some degree of conserved plastic response in the population. These genes also did not exhibit significant Gene Ontology (GO) enrichment.

To further understand how the global patterns of gene expression plasticity varies among different genetic clusters, we analyzed genome-wide expression profile in multivariate space by implementing DAPC. The linear discriminate function was derived to maximize the variation between genetic clusters with respect to their native habitats (Inland genotypes at inland site vs. Coastal genotypes at coastal site), and transplants samples were projected on that discriminant axis to estimate expression plasticity of a focal genetic cluster (fig. 2*E*: DAPC plot). We observed that the magnitude of expression plasticity varied between different genetic clusters and each pairwise comparison (significant at each pairwise comparison level, adjusted *P* < 0.05). The Coastal genetic cluster demonstrated the least plastic transcriptome response compared with any of the inland clusters.

### Genetic Architecture of Expression Divergence

Previously, we studied expression divergence between inland and Coastal ecotypes of *P. hallii* using eQTL analyses in a field drought experiment (Lovell et al. 2018). In that study, we discovered a predominance of *cis*-regulatory divergence, along with several *trans*-QTL that were enriched for drought responsiveness. Here, we leveraged our more expansive study of inland and coastal expression diversity to test the generality of these results. In particular, we ask whether differentially expressed genes (DEGs) detected in the current study (G, E, or G×E) were enriched for the occurrence of inland/coastal eQTL (*cis*, *trans*, *cis*-eQTL × drought, *trans*-QTL × drought) (fig. 2*F*) from our earlier study. We observed an overrepresentation of *cis*-eQTL and *cis*-eQTL × drought in G genes, along with an overrepresentation of *cis*-eQTL × drought genes in our G×E lists. Interestingly, we observed a significant underrepresentation of *cis*-eQTL genes in our E and G×E categories and a significant underrepresentation of *trans*-QTL × drought genes in our G×E genes. Overall, we found more overlap among studies in terms of *cis*- and *cis*-eQTL × drought than for *trans*-factors.

### Detecting Selection on Gene Expression Divergence From Reciprocal Transplantation

**Fig. 3.**
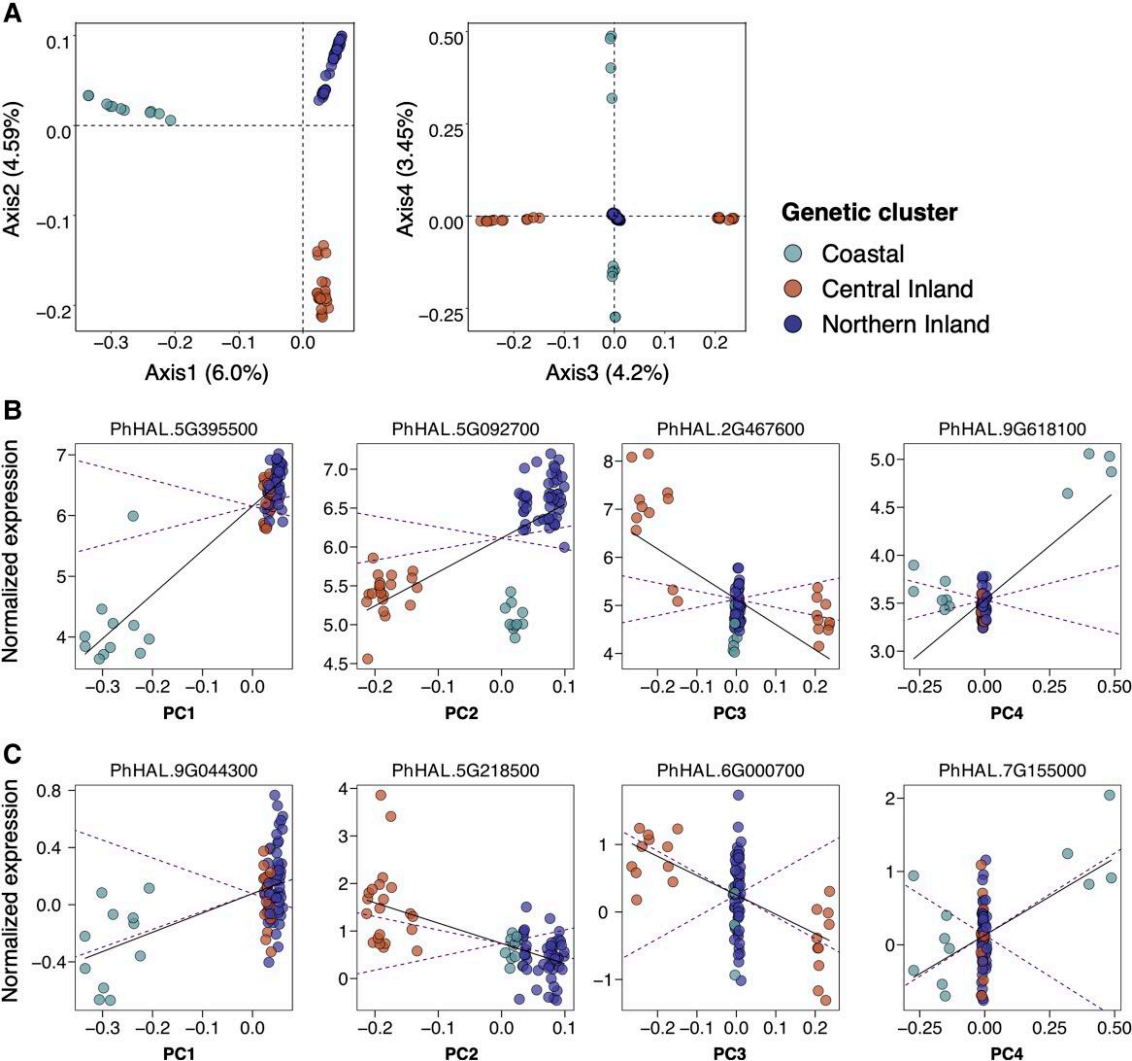
Outlier gene expression for reciprocal transplantation based on *Q*_PC_ analysis. (*A*) The first four genetic PCs of the kinship matrix. Each circle represents different accessions which are colored by three different genetic clusters. Each axis is labeled by the percentage of variation explained by that PC compared with the total variation. (*B*) Genes that showed evidence of selection on their CDE. The mean-centered expression of the genes averaged across two sites was plotted against the eigen value of corresponding PCs for selected genes that were identified as outliers from neutral expectation (please refer to the main text, supplementary tables S8 and S10, and Supplementary Material online, for detailed annotation of genes). Each point represents a single *P. hallii* natural accession and is colored by genetic cluster. The linear regression is plotted as a solid line, and the 95% CIs of the neutral expectation are shown as dotted lines in each plot—expression values outside of the CI envelope are taken as putatively adaptive divergent expression. (*C*) Genes that demonstrated PDE between inland and coastal sites. The data format is the same as described for *B* except the mean-centered expression of the genes difference across two sites (coastal–inland) was plotted against the eigen value of corresponding PCs.

Given the considerable gene expression divergence present among *P. hallii* populations, we subsequently asked to what degree the observed divergence in gene expression and its plastic response to the environment is the result of neutral or adaptive evolutionary processes. To infer the past action of natural selection, we implemented the *Q*_PC_ method. This method uses PCs of relatedness matrix to estimate additive genetic variance and test for departure from neutral expectations in trait divergence. Figure 3*A* demonstrates how the lower-order PCs separate the major genetic clusters of *P. hallii* across the axes on which the expression divergence was tested. The neutral expectation was derived from estimated additive genetic variance from higher-order PCs. Figure 3*B* and *C* delineated examples of expression traits evolving neutrally and under selection (excessive divergence), respectively, on the first PC which separated the Coastal genetic cluster from inland clusters. As mentioned above, we noticed a remarkable variability in the pattern of historical recombination across the *P. hallii* genome, with likely important consequence for evolutionary dynamics in the recombining and nonrecombining portions. As such, we employed the *Q*_PC_ method in two different modes to obtain a constitutive component of expression divergence: 1) a relaxed mode where we applied the *Q*_PC_ method on the mean expression of all expressed genes (18,773) across sites and 2) a constrained mode on the mean expression of genes which resided in the highly recombined regions (15,735) across sites. Similarly, we explored the plastic component of expression divergence by studying the difference in expression between field sites for each accession. We designate these patterns as constitutively divergent expression (CDE) and plastic divergent expression (PDE).

For CDE, we found 1,006 and 514 unique genes with excessive divergence beyond neutral expectation for the first 5 PCs under the relaxed mode and constrained mode, respectively (false discovery rate [FDR] threshold < 0.1). While implementing the relaxed mode analysis, we observed an excess of genes with constitutively elevated expression divergence from the low recombined regions compared with the expressed background genes (Fisher's exact test odd ratio 5.4; *P* < 2.2e^−16^). It is possible that this elevated expression divergence is the result of ineffective purifying selection as might be expected in genomic regions of low recombination. Given our interests were primarily in identifying putatively adaptive candidate genes by comparing with a genomic background, we were concerned with the impacts of including the nonrecombining pericentromeric regions in our comparisons. Therefore, from here onward, we only considered the constrained mode analysis for both CDE and PDE. In the CDE category, PC1 accounted for almost half of these genes (224) (supplementary table S8, Supplementary Material online). GO enrichment analysis of these genes identified ten GO terms including terms related to response to stimulus and ion binding (supplementary table S9, Supplementary Material online; nominal *P* < 0.05). PC2 accounted for 161 genes with an enrichment of 19 GO terms such as pollen–pistil interaction and response to stress. PC3, PC4, and PC5 accounted, respectively, for 96, 50, and 65 genes which exhibited significant divergent expression. CDE genes on PC3, PC4, and PC5 were enriched with GO terms including terms related to anion binding and endopeptidase activity, cellular protein metabolic process and ATPase-coupled transmembrane transporter, and RNA processing and chromosome segregation, respectively. Figure 3*B* depicts the expression profile of a few example genes along the corresponding PCs for which nonneutral expression divergence has been inferred. For instance, PhHAL.5G395500 (high-affinity potassium transporter 1) demonstrated expression divergence along PC1 between coastal and inland populations (supplementary table S8, Supplementary Material online). We have detected 82 genes that show evidence of selection on multiple PCs indicating that adaptive expression divergence could be a function of divergence across more than 2 genetic clusters. For example, PhHAL.5G092700 (oxidoreductase family protein) demonstrated excessive expression divergence for both PC1 and PC2. PhHAL.2G467600 (AP2 domain-containing protein) and PhHAL.9G618190 (heat shock protein 60) were plotted as examples of divergently expressed on PC3 and PC4, respectively (fig. 3*B*).

For the PDE category, we failed to detect any statistically significant signal of nonneutral divergence that passed multiple testing correction. However, we detected 29 genes that passed the nominal *P* value threshold (<0.05) (supplementary table S10, Supplementary Material online). The majority of these were detected along PC2 (11 genes) and were related to plastic expression divergence between the Central compared with the other two genetic clusters (fig. 3*C*). Only three genes were detected with significant divergence between the inland and Coastal genetic clusters along PC1. Nine and four genes were detected along PC3 and PC4 between the central and northwestern inland genetic clusters. We plotted PhHAL.9G044300 (galastosyl transferase family protein), PhHAL.5G218500 (chitinase A), PhHAL.6G000700 (cysteine proteinase superfamily protein), and PhHAL.7G155000 (ferric-chelate reductase) as examples of plastic expression divergence between populations along PC1, PC2, PC3, and PC4, respectively (fig. 3*C*).

Overall, we detected strong evidence for adaptive constitutive gene expression divergence (514 CDE genes) and weaker signal for adaptive plastic gene expression (PDE) divergence in our study. Moreover, we observed enrichment of both *cis* (odd ratio 1.7, Fisher's exact test *P* = 9.7e^−8^) and *cis*-G×E eQTL genes (odd ratio 1.7, Fisher's exact test *P* = 3.2e^−4^) but no enrichment of trans and *trans*-G×E eQTL in our CDE category.

### Relationship between Expression and Sequence Divergence

**Fig. 4.**
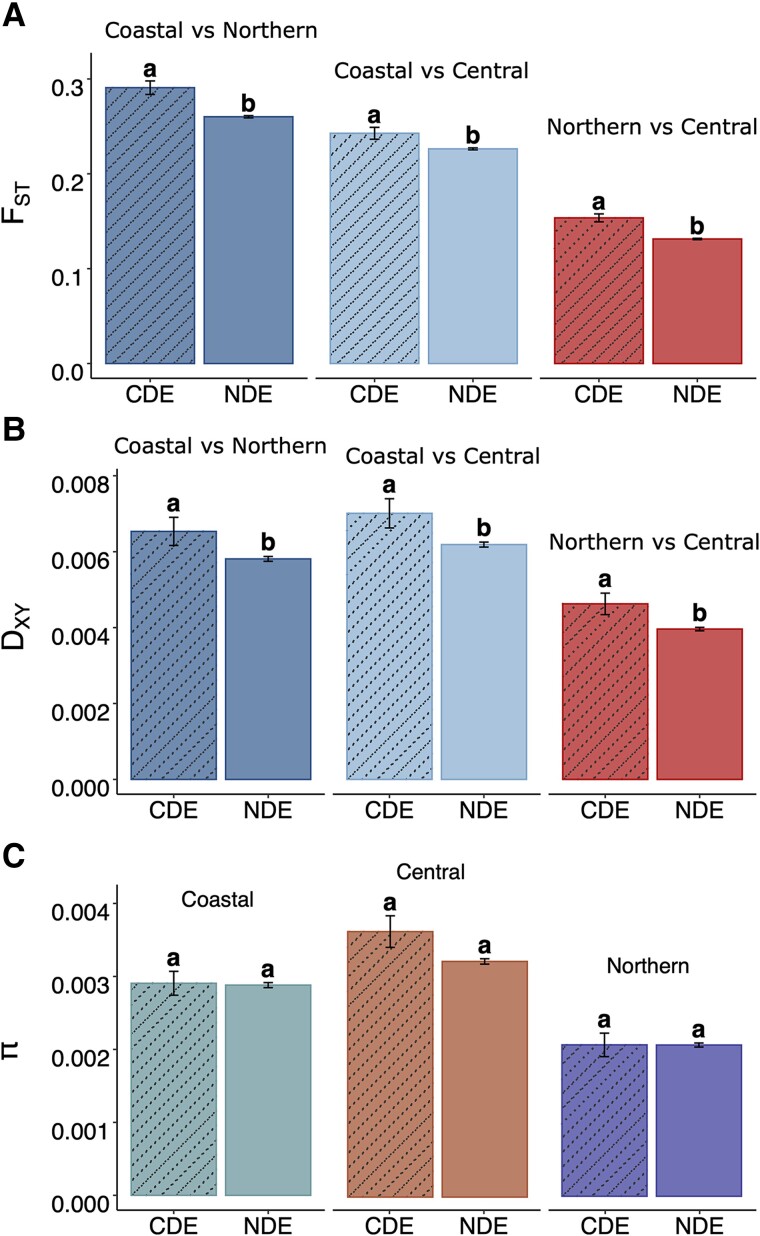
Divergence statistics at CDS of CDE and nonsignificant divergent expression (NSE) genes (*A*) relative fixation index (*F*_ST_), (*B*) absolute divergence (*D*_XY_), and *C*) nucleotide diversity (pi). The vertical line centered at the mean indicates standard error (SE).

In order to understand the relationship between expression divergence and sequence evolution, we compared patterns of polymorphism and divergence between genes with putatively adaptive CDE and a set of background genes (15,237 expressed genes with nonsignificant divergence [NDE]). Given the relatively weak signal for PDE genes, we chose to exclude these from our analyses. Here, we looked at within and between genetic cluster diversity at the different annotated gene features including coding sequence (CDS), intron, and 3′ and 5′-untranslated regions (UTR). To explore patterns of diversity and divergence in putative regulatory sequences, we also studied proximal promoters (2 Kb upstream from transcription start site) of target gene models. We observed significantly higher relative divergence (*F*_ST_) at CDE genes compared with NDE genes for all three possible pairwise contrasts at each gene feature separately (fig. 4*A*; supplementary table S11, Supplementary Material online).

To address whether genetic variation between or within populations drives this pattern of differentiation, we estimated absolute divergence (*D*_XY_) and nucleotide diversity (π) statistics at each of the gene features. We observed significantly higher absolute divergence (*D*_XY_) for all three contrasts for CDE genes compared with NDE at CDS, 5′-UTR, and promoter regions (except Northern vs. Central contrast at 5′-UTR and promoter) (fig. 4*B*; supplementary table S11, Supplementary Material online). However, there was no difference in *D*_XY_ at intron and 3′-UTR between CDE and NDE category genes in any pairwise contrast. No significant difference in nucleotide diversity (π) within each genetic cluster was observed except for Northern and Central inland clusters at promoter regions (fig. 4*C*; supplementary table S12, Supplementary Material online). Overall, we observed excessive relative and absolute divergence at CDS and promoter regions for CDE genes compared with NDE genes.

### Tests for Positive Selection for Proteins with Divergent Expression

Since we observed higher relative and absolute sequence divergence among populations for the protein CDS of CDE genes, we hypothesized that the proteins of these genes might exhibit different rates of evolution among *P. hallii* lineages. Therefore, we quantified the ratio of substitution rates for nonsynonymous and synonymous sites (dN/dS) of all one-to-one orthologous gene pairs of Coastal and Northern Inland lineages based on the existing annotated reference genomes. In total, we evaluated 184 CDE genes. We observed significantly higher evolutionary rates for CDE genes compared with NDE genes (Mann–Whitney *U* test *P* < 0.01). Downsampling the NSE category to the number of CDE category also revealed a higher evolutionary rate for genes in the CDE category (Mann–Whitney *U* test *P* < 0.01). We also detected a negative correlation between both dN/dS and dN and average expression level across site (*P* value of Spearman's rank correlation test <0.05). Therefore, we further tested for partial correlation of constitutive divergence expression with dN/dS and dN while controlling for mean expression and detected a positive correlation for both the factors (CDE genes had higher dN/dS and dN; *P* value of Spearman's rank correlation test <0.05). Similarly, mean expression level of CDE genes was lower than NDE genes (Mann–Whitney *U* test *P* < 0.01). However, we did not find any difference for the frequency of genes with dN/dS >1 for the CDE group compared with NDE group (χ^2^ test *P* = 0.66) which could be likely candidates for positive selection. Specifically, we detected 4 and 129 genes in CDE and NSE categories, respectively, with estimated dN/dS >1.

Finally, to identify genes experiencing positive selection in the *Panicum* lineage, we compared the likelihood of PAML's (version 4.9) site-specific selection model (M2a) with that of a neutral model (M1a) across the *Panicum* phylogeny (Yang 2007). Here, we included the two subgenomes (N and K genomes) of *P. virgatum* to infer ancestral states and restricted our analysis to 7,419 CDE genes with one-to-one ortholog pairs for all 6 pairwise comparisons. After correcting for multiple tests, we detected 163 genes which exhibited evidence of positive selection (supplementary table S13, Supplementary Material online). However, this set of positive selection candidates was not enriched for CDE genes (χ^2^ test *P* = 0.66).

## Discussion

### Patterns of Gene Expression Divergence in *P. halli* Population

In this study, we decomposed the heritable component of constitutive and plastic transcriptome-wide gene expression divergence in *P. hallii* using a reciprocal transplant experiment at two native sites. In congruence to earlier studies (Whitehead and Crawford 2006a; Staubach et al. 2010; Groen et al. 2020), most of the expressed genes in our study have evolved (nearly) neutrally. Noticeably, we detected relatively strong signals of constitutive expression divergence beyond the expectation of neutral evolution for several hundred genes (514 genes) and only a weak signal of putatively adaptive plastic expression divergence. Constitutive expression divergence (Hodgins et al. 2016; Fuess et al. 2021) and population-dependent plastic responses of transcripts to changing environment or treatment (Kenkel and Matz 2016; Mäkinen et al. 2017) have been reported in many study systems. However, heritable expression divergence can also result from random genetic drift, but only a handful of studies have implemented an analysis framework to incorporate a neutral model to detect the effect of natural selection. One example is a study by Whitehead and Crawford (2006b) on teleost fish populations which inferred that expression divergence of several genes in a common garden experiment was due to natural selection. Groen et al. (2020) estimated stronger selection on gene expression in drought conditions compared with wet conditions and reported that rice accessions which were more plastic experienced fitness benefits. Adaptive plastic expression response to changing environments can provide resilience to a population and therefore is of central interest, especially in the context of recent climate change. In this study, we observed a weak signal of such responses in *P. hallii*. Several factors such as high cost of plasticity, lack of genetic variation toward plastic response, low gene flow, relaxed selection due to environmental variability, or maladaptive plasticity can hinder the process (Crispo 2007; Murren et al. 2015). Hypothetically, the cost of plasticity may lead to CDE of genes across diverse habitats and selection can reinforce variability when it is heritable and gene flow is weak. Our reciprocal transplantation framework allowed for the identification of the constitutive and plastic components of expression divergence and the evaluation of factors that might constrain and/or facilitate this divergence in the context of ecological variability. For instance, we found *CPK13* (*P. hallii* ortholog of *Arabidopsis thaliana* AT3G51850.1 *calcium-dependent protein kinase 13*, *PhHAL.2G319400*) and *PHS1* (*P. hallii* ortholog of *A. thaliana* AT5G23720.1 *propyzamide-hypersensitive1*, *PhHAL.5G392700*) were constitutively highly expressed in the leaf of both inland genetic clusters compared with the Coastal cluster. These genes were previously detected as *cis*-eQTL candidates by Lovell et al. (2018) (supplementary table S8, Supplementary Material online). CPK13 controls stomatal behavior by regulating the expression of guard cell potassium channels (KAT1 and 2), whereas PHS1 controls stomatal behavior via abscisic acid (ABA) signaling. The constitutive expression of CPK13 and PHS1 may provide fitness benefits for *P. hallii* that are native to water-deficit inland habitats, but it may have a trade-off in wet coastal habitats due to the high cost of expression.

### Recombination Landscape Delineates Excess of Constitutive Expression Divergence in Low-Recombining Regions

Recombination can generate novel allelic combinations by bringing together multiple beneficial alleles or decoupling them from deleterious alleles. Recombination rate can vary between different species, genetic clusters within species, or even along chromosomes. Grass species such as rye (Schreiber et al. 2022) and barley (Dreissig et al. 2019) show striking variation of recombination rate along the length of their chromosomes. In the same line of these reports, *P. hallii* exhibits remarkable variation in the recombination landscape across chromosomes, leaving large parts of the chromosome near pericentromeric regions as mostly nonrecombining (fig. 1*C*; supplementary fig. S2, Supplementary Material online). The genomic landscape of recombination can play a key role in shaping the pattern of diversity along the genome by altering the efficacy of selection. Indeed, a different pattern of expression divergence across the recombination landscape was observed. We detected that divergently expressed genes (both G category genes detected in factorial model and CDE genes detected by outlier test) were enriched in genomic regions with lower recombination rates. However, plastic genes (G×E) were significantly depleted in these regions. Excessive constitutive expression divergence associated with these regions could be an indicator of maladaptive expression divergence; it could arise due to inefficient purifying selection and the accumulation of deleterious mutations. Expanding our test for positive selection on protein evolution including genes in the low-recombining regions detected no excess of positive selection (*P* < 0.78) further supporting this idea.

### Association of Expression Divergence with Sequence Evolution

Different trends of association between protein evolutionary rate and expression divergence were observed among different model systems and taxa (Moyers and Rieseberg 2013; Nuzhdin et al. 2004; Liao and Zhang 2006; Renaut et al. 2012; Hodgins et al. 2016). We found significant elevated relative (*F*_ST_) and absolute divergence (*D*_XY_) among genetic clusters and faster protein evolution (dN/dS) for CDE genes relative to a genomic background of NDE genes (expressed but lacking expression divergence). Increased fixation rates due to positive selection on CDE genes reduced purifying selection on these genes in at least one genetic cluster, or both might lead to this relationship. However, we did not observe an excess of CDE genes under positive selection. Therefore, we reason that many CDE genes might have evolved under relaxed purifying selection. Alternatively, the relatively deep divergence time between *P. virgatum* and *P. hallii* (∼6.6 Ma) may result in lower power for detecting selection events across *P*. *hallii* lineages.

Studies in both animal and plant systems have detected that gene expression level was associated with sequence evolution and genes with higher expression level evolve more slowly (Duret and Mouchiroud 2000; Hodgins et al. 2016). This could be due to the fact that highly expressed genes tend to interact with a wide range of partners, and therefore are less likely to tolerate mutation (Duret and Mouchiroud 2000); or they could be under purifying selection due to fitness loss from protein misfolding as a consequence of mutation (Wu et al. 2022). We similarly detected a negative correlation between mean gene expression and the rate of protein evolution (dN/dS). Moreover, our analysis revealed lower mean expression level for CDE genes in comparison with NDE genes. This could be attributed to moderately expressed genes that are less ubiquitous and consequently had fewer pleiotropic effects. This scenario might have led to the evolution of population-specific expression patterns, thereby conferring fitness advantages within native habitats. Moreover, the cost of plasticity, trade-offs in foreign environments, or lack of gene flow could allow selection to reinforce the difference in expression among genetic clusters and eventually lead to constitutive divergence. It is likely that the very diverse habitats of inland and coastal *P. hallii* have limited gene flow (even in sympatric habitats), along with extensive inbreeding, and might have contributed to the abundance of constitutive divergence.

### Enrichment of *cis*-eQTLs for Putatively Divergent Gene Expression

*Cis*-regulatory variants have been reported to contribute a greater proportion of expression variation in many plants and animal model systems compared with *trans*-variants (Schadt et al. 2003; Guo et al. 2004; Springer and Stupar 2007; Lovell et al. 2016; Lovell et al. 2018), and it has been hypothesized they may persist over longer evolutionary time scales because they incur fewer deleterious pleiotropic effects (Prud'homme et al. 2007; Stern and Orgogozo 2008). In this study, we observed an enrichment of both *cis*- and *cis*-G×E (*cis*-x drought) eQTLs detected by Lovell et al. (2018) in our putatively divergent expression category detected by either factorial models (G genes) or CDE genes from *Q*_PC_ outlier tests. This enrichment of *cis*-eQTL and higher divergence at the promoter region of CDE genes suggest that *cis*-variants might play an important role in expression divergence in *P. hallii*. *Trans*-variants are expected to be more pleiotropic (Wittkopp 2007 but also see Lynch and Wagner 2008), since these interact with a wider range of target genes and hence are likely to have a more deleterious effect than *cis*-variants. Hence, it is likely that *cis*-variants, which exhibit fitness trade-offs between different habitats of *P. hallii*, would be ideal candidates for expression divergence and could be further reinforced by natural selection when gene flow is limited between genetic clusters.

Broadly, reciprocal transplantation experiments integrated with a *Q*_PC_ outlier framework allowed us to identify several hundred genes with putatively adaptive constitutive expression divergence but only a weak signal for adaptive plastic divergence. This suggests adaptive plastic responses are rare or were undetected in our experimental context. We observed elevated sequence divergence of promoters and CDS of constitutive expression outliers, yet found little evidence for positive selection on these proteins. Furthermore, enrichment of genes with constitutive expression with *cis*-eQTL genes strengthens the hypothesis that *cis*-regulatory divergence is a primary driver of expression variation in *P. hallii.* Together, our study provides a unique look at the evolutionary forces driving patterns of expression polymorphism and divergence in a native grass and identifies outlier candidate genes for future functional genomic studies.

## Methods

### SNP Calling and Filtering

Details for the field collections of *P. hallii* are provided in Palacio-Mejía et al. (2021) and describe collection campaigns from natural field sites distributed across southwest United States (fig. 1*A*; supplementary fig. S1*A* and table S1, Supplementary Material online). The geographic range of the *P. hallii* var. *hallii* is larger compared with *P. hallii* var. *filipes*, and thus, the sample size is greater for the former (75 var. *hallii* and 11 var. *filipes*). *Panicum hallii* is a strongly selfing species (mean inbreed coefficient 0.89), and field collected material is naturally inbred and highly homozygous (Lowry et al. 2015). Some of these accessions and resequencing data were included in previous studies to investigate genetic variation and population structure (Gould et al. 2018; Lovell et al. 2018). Here, we include 11 newly resequenced accessions (supplementary table S1, Supplementary Material online). In order to call SNP variants, we first filtered raw reads for each genotype using BBDuk program from BBTools suite (Bushnell et al. 2017; Bushnell 2020) with a minimum average quality of 20. Next, quality trimmed reads were mapped against the *P. hallii* var. HAL2 reference genome v2 (https://phytozome-next.jgi.doe.gov/info/PhalliiHAL_v2_1) using Burrows–Wheeler Alignment (BWA) mem algorithm (Li and Durbin 2009) with default parameters. We chose the HAL2 reference genome over FIL2 since the HAL2 reference has near complete chromosome-level assembly with the largest N50 and fewest unassigned scaffolds at the chromosome level (Lovell et al. 2018). Moreover, sample sizes for var. *hallii* are much larger compared with var. *filipes*. We further filtered alignments with the SAMtools software package (Li et al. 2009) with a minimum mapping quality of 20. Filtered alignments were sorted by coordinates, duplicated reads were marked, read groups were assigned for individual genotypes, and finally, alignments were indexed by Picard tool (v2.20.4) (http://broadinstitute.github.io/picard/). Alignments around the insertion/deletion regions were masked by the IndelReligner tool, and the first iteration of SNPs was called by the HaplotypeCaller algorithm from the Genome Analysis Toolkit (GATK) version 3.8.1 (McKenna et al. 2010). Later base recalibration was performed using a set of high-quality variants by the BaseRecalibrator function, and for the second iteration of variant calling, we used the “--emitRefConfidence GVCF” flag to obtain confidence scores for each site in the genome irrespective of variants or not. Finally, we used GenotypeGVCFs program to collect variant calls and confidences across all individuals and produce genotype calls for each site by setting the “-allSites” flag. We then selected SNP variants and filtered for SNP calling based on the hard filtering recommendation by GATK (https://github.com/tahia/SNP_calling_GATK). SNPs which passed these quality filters were retained as high-quality SNP variants for this diversity panel. Variant sites were further filtered sequentially to keep 1) only biallelic variants, 2) any genotype with DP ≥3 was kept else masked as missing, 3) sites kept which have percent of heterozygosity <3.6 (85th percentile of the distribution), and 4) <20% missing data and finally obtained 23,692,316 high-quality biallelic SNPs. Nonvariant sites were separated by VCFtools (Danecek et al. 2011) and filtered by read depth, quality, and percentage of missing individuals (--minDP 20 –minQ 30 –maxmissing 0.8).

### Analysis of Population Genetic Variants and Structure

As with previous studies that included the presence of highly divergent groups (e.g., subspecies and ecotypes), population genetic structure was first assessed hierarchically (Walsh et al. 2017; Napier et al. 2019; Lovell et al. 2021). Specifically, due to the strongly differentiated ecotypes present in our study system, we first identified the broadest genetic population structure using DAPC in the adegenet (v2.0.1) package (Jombart et al. 2010; Jombart and Ahmed 2011). As this method is not based on common assumptions underlying many population clustering approaches (e.g., Hardy–Weinberg equilibrium and linkage equilibrium), it is a valuable tool for examining broad structural divisions. High-quality SNPs were LD pruned (--indep 50 5 2) using plink (Purcell et al. 2007) and annotated by SnpEff tool (Cingolani et al. 2012) with the HAL2 reference genome annotation. From the SNP annotation, synonymous SNPs in CDS were selected as putatively neutral variants and were further filtered for minor allele count and missing data (<10% missing data or minor allele count <3). We used a random set of 50,000 of these synonymous SNPs for genetic cluster analysis. Prior groups were determined by first transforming the genetic data using PCA, and then, the first ten PCs were used in a k-means algorithm to classify individuals into the broadest two possible groupings aiming to maximize the variation between groups. DAPC was then implemented using ten retained principal components (PCs) to provide a description of the genetic clusters (i.e., the two ecotypes).

After classifying individuals into two broad genetic groupings consistent with the ecotypes, we evaluated the genetic structure of and potential admixture between individuals belonging to the more well-represented *hallii* group using the Bayesian clustering algorithm implemented in STRUCTURE (v2.3.4) (Pritchard et al. 2000; Falush et al. 2003). Specifically, the same set of loci were used, but we subset the individuals to those with a DAPC posterior probability of >0.95 for belonging to the *hallii* genetic subpopulation. Within STRUCTURE, we utilized an admixture model with correlated allele frequencies and no prior information. The analysis entailed 20,000 burn-in steps and 100,000 replicates of 1–8 genotypic groups (*K*), each of which was run 10 times. Using STRUCTURE HARVESTER (Earl and vonHoldt 2012), the resulting output was compiled and evaluated for the optimal *K* value based on Δ*K* (Evanno et al. 2005). One accession (COL) which demonstrated a small degree of admixture between two inland genetic clusters was removed for further analyses.

### Estimation of Population-Scaled Recombination Rate

We used the high-quality imputed SNP calls of three different genetic clusters to estimate recombination rate (ρ/kb, ρ = 4Ne × re, where Ne is the effective population size and re is the effective recombination rate per generation in a population). To account for different sample sizes of each genetic cluster, we randomly subsample individuals from each population (*n* = 11) for this study. First, SNPs were filtered with the following criteria: 1) all heterozygous calls were masked as missing, 2) SNPs with <5% missing data were retained, and 3) minor allele frequency > 0.1 was retained and 4) thinned by 50 base pairs (bp) distance. Subsequently, SNPs were imputed by Beagle V5.4 (window = 5, overlap = 2, iterations = 30, err = 0.001, burn-in = 10; Browning et al. 2018). The interval program of LDhat package (Auton and McVean 2007) was used to estimate the ρ/kb for each nonoverlapping 5 Mb intervals (theta 0.001, -its 60,000,000 -samp 5,000 -bpen 5). We used the stat program of the same package to summarize ρ/kb values, and the first 50,000 iterations were removed as burn-in. Later, mean recombination was estimated over 1 Mb interval with a step size of 100 Kb. While comparing recombination landscapes across different genetic clusters, ρ values were scaled within each cluster by dividing by the maximum value. The minimum overlapping contiguous regions with elevated recombination rate across genetic clusters have been defined as highly recombined regions (supplementary table S2, Supplementary Material online). We used a RIL mapping population generated from inland and coastal references (Haque et al. 2022) to estimate the correlation between recombination rate and crossover evens. The number of crossover events was estimated in 1 Mb intervals with a step size of 100 Kb using the *find.breaks* function from “xio” package (Broman et al. 2002).

### Field Sites and Reciprocal Transplantation

We carried out a reciprocal transplant experiment in 2017 at two different field sites: one representing an inland habitat (The Brackenridge Field Laboratory of the University of Texas at Austin, Austin, TX, USA: 30°15′53.928″N, 97°44′47.7492″W; hereon referred as BFL) and another representing a coastal habitat (USDA Plant Materials Center, Kingsville, TX, USA: 27°30′57.456″N, 97°56′34.134″W; hereon referred as KINGS) (supplementary fig. S4*A* and *B*, Supplementary Material online). The distance between these inland and coastal field sites is ∼232 miles (374 km). The two field sites have varied soil composition in terms of available soil nutrients and soil conductivity. The soil at the coastal site contained more cations and higher electrical conductivity than the soil at the inland site (supplementary fig. S5*A* and *B*, Supplementary Material online). The mean temperatures at the coastal and inland sites (March to June) were 25.1 °C and 23.1 °C, respectively, and cumulative precipitation is relatively higher at the coastal site compared with inland site (supplementary fig. S5*C*, Supplementary Material online).

All accessions were grown from seed in a greenhouse (14 h days at 500 μE m −2 s −1, 28 °C; 10 h nights at 24 °C) located at the Brackenridge Field Laboratory in Austin Texas. On March 8, seeds were scarified with sandpaper in order to remove the seed coat. Scarified seeds were placed on wet sand in 25-mm-deep Petri dishes which were then sealed with parafilm, moved to a greenhouse bench, and randomly cycled to standardize environmental conditions. On March 15, seedlings were transplanted to 2.5-inch pots (SVD-250-BK, T.O. Plastics, Clearwater, MN, USA) filled with soil (60:40 mixture of Promix BX, Premier Tech Horticulture, Riviere-du-Loup, Quebec, Canada, and Turface, Profile Products, Buffalo Grove, IL, USA) and arranged in 32 cell trays. On March 25, seedlings were thinned to leave one plant per pot, and at least six healthy individuals per accession were selected for field transplantation. Plants were further randomized within trays into the field experiment layout. Trays were then randomly cycled in the greenhouse once every week and irrigated as needed.

Our experimental design consisted of 86 *P. hallii* accessions grown at two field locations with a minimum of three biological replicates. Field transplantation was performed on April 7 at the inland site and April 12 at the coastal site. Soil at each planting site was tilled to a depth of 6 in with a roto-tiller, and weed barrier fabric (Sunbelt 3.2 oz, Dewitt Company, Sikeston, MO, USA) was applied to the planting area for weed control. Holes large enough to accommodate the transplants and their expected growth were cut in the weed fabric in a honeycomb fashion. Three individuals from each accession were reciprocally transplanted to the field in a randomized design at each field site. The inland site was 10 individuals wide and 84 individuals long, and the coastal site was 21 individuals wide and 37 individuals long, with a spacing of 0.75 m between individuals. Plants were carefully pulled out from the pots along with soil and sunk directly into the ground. A border row of *P. hallii* surrounded both field sites and was used to control for edge effects.

### Tissue Collection for RNA

Tissue collection for RNA and phenotypic measurements took place on June 14 at the inland site and June 20 at the coastal site between 9:30 Am and 12:15 Pm. Both collection days had mostly clear skies, with mean local temperatures during collection periods of 29.55 °C at the inland site and 30.28 °C at the coastal site. Tissue collection for RNA and RWC was done simultaneously at the time of collection. The tissue collection for RNA was standardized across the genotypes and sites by collecting fully expanded flag leaves from each plant. All plants were at the reproductive stage during the time of tissue collection. In brief, a leaf was excised from the plant and immediately chopped into pieces (∼1–2 cm) in 2-mL Eppendorf tubes loaded with three stainless beads. Samples were flash-frozen in liquid nitrogen and moved to a −80 °C freezer.

### RWC Measurement and Statistical Analysis

For RWC measurements, two leaves were cut at the ligule and taken from each plant during each sampling period. For particularly small plants, a third leaf was harvested. Fresh weight of leaves was recorded and the cut petiole end of each leaf was then placed into a 15-mL Falcon tube, with roughly 5 mL of water, and both leaves were held in place with a cotton ball. Leaves were then stored in a dark cooler while in the field and transferred to a refrigerator, for a total of ∼ 8 h to reach full rehydration. The turgid weight was then recorded, and the leaf tissue was subsequently dried in a 65 °C oven for 24 h to record the dry weight. RWC was calculated as (fresh weight − dry weight)/(turgid weight − dry weight) × 100. We analyzed RWC using a two-way ANOVA model testing for the effect of genetic clusters (G), field environment (E), and their interaction (G×E). The effect of genetic clusters was tested separately by site using one-way ANOVA, and pairwise comparisons between the means of genetic clusters were computed using Tukey honestly significant difference (HSD).

### RNA Extraction and Library Preparation for Sequencing

Leaf tissue was ground in to a fine powder using Geno/Grinder 2010 (SPEX SamplePrep. Metuchen, NJ). RNA extraction was performed according to the TRIzol Reagent user guide (Cat # 15596018). In brief, the ground leaf tissue powder was homogenized in 1 mL of TRIzol reagent and then incubated with 200 µL of choloroform:isoamyl alcohol 24:1 (Sigma, C0549) for 10 min on rotator mixer at room temperature followed by centrifugation. The clear supernatant was then transferred to a new 1.5-mL tube and added an equal volume (*V*/*V*) of isopropanol to precipitate nucleic acid. Samples were mixed well and the nucleic acid pellet was obtained by centrifugation. The pellet was washed with 75% alcohol, air dried for 10 min at room temperature, and suspended in 30 µL of 10 mM Tris-Cl (pH 8.0) to obtain RNA. The RNA samples were treated with DNase I (Ambion, AM2222) to remove contaminating genomic DNA. RNA concentration was measured using NanoDrop ND-1000 spectrophotometer.

We constructed RNA-seq libraries using a high-throughput 3′-mRNA TagSeq 3′-tagseq approach (Meyer et al. 2011; Lohman et al. 2017; Weng and Juenger 2022). TagSeq is widely used for constructing libraries directed at the 3′-end of mRNA fragments enriched in a size range of 300–500 bp. Briefly, 1 µg of RNA from each sample was incubated at 70 °C to achieve fragmented RNA molecules of desired range. The entire fragmented RNA was used in first-strand cDNA synthesis using RNA oligo primer (S-Ill-swMW) and cDNA amplification. Further, 50 ng of purified cDNA was barcoded using Illumina-specific barcodes (ILL-BC) and multiplexed using Illumina TruSeq universal adapters (TruSeq-Un). An equal volume of 30 randomly chosen libraries were pooled together and ran on agarose gel to excise the fragment in the 400–500 bp size range. The resultant library pool was loaded on one lane of an Illumina HiSeq 2500 analyzer at the HudsonAlpha Genome Sequencing Center, Huntsville, AL, USA. We recovered between 5 and 7 million 100 bp single end (SE100) reads per sample.

### TagSeq Sequence Data Processing

In total, we sequenced 508 TagSeq libraries from samples collected from replicated accessions grown at our inland and coastal field sites. A few samples were lost due to poor RNA extraction or library construction failure. On average, we sequenced 6 libraries from each accession (inland, 249, and coastal, 259). We used FastQC to evaluate the sequencing quality (FASTQC 2017) for various quality matrices such as average sequence quality, GC content, k-mer profile, and presence of adapter sequences. These matrices were used for finding optimum parameters for quality filtering of raw sequences. We trimmed the first nine bases (including degenerate bases) from the sequence adapter and homopolymers (polyA or polyT) for base count of ≥15 mer using Cutadapt (Martin 2011). We allowed reads with an average sequencing quality of ≥20 and retained minimum sequence length of 70 bases. We generated several k-mer profiles with varied k-mer lengths for the *P. hallii* transcriptome and asked what percentage of the total transcriptome can be detected uniquely with that specific k-mer length across the total length of all transcripts. This analysis showed that ∼82% and ∼90% of unique transcripts can be detected by k-mer length of 50 and 100 bp, respectively. Therefore, we chose 70 bases as the minimum sequence length to detect ∼85% of unique transcripts for this genome. Filtered reads were then mapped to *P. hallii* var. *halli*i HAL2 reference genome (v2.0) using BWA mem algorithm (Li and Durbin 2009). Mapped reads were further filtered using SAMtools, and the reads with mapping quality ≥10 were retained. Duplicated alignments were marked by Picard (http://broadinstitute.github.io/picard/). We used FeatureCounts (Liao et al. 2014) to generate an expression count matrix from filtered and duplication marked alignments using the existing annotated gene models of *P. hallii*. Only first-strand–specific reads were considered as a count that had fragment length ≥70 bp (--readExtension3 0 –readExtention5 0 -d 50 -s 1). Sequencing results and mapping statistic are given in supplementary table S14, Supplementary Material online.

### Differential Gene Expression Analysis

We detected gene expression for 18,773 genes from 33,263 annotated gene models of the HAL2 reference genome. To improve DEG detection sensitivity, we first removed genes that had low expression by filtering those with an average count <1 across all 508 samples. We used DESeq2 package in R for DEG analysis (Love et al. 2014). Libraries were first normalized for their size and gene counts were transformed by variance stabilizing transformation (VST) using the fitted dispersion–mean relationship. We used 1,000 genes with the highest variance after normalization and transformation in the PCA to visualize the global variation in gene expression between samples.

For DEG analysis, we used DEseq2 to fit a factorial model to test for the effect of genetic cluster/origin (G), field environment/transplant site (E), and their interaction (G×E). Note that our statistical approach had lower power to detect interaction terms (G×E) compared with main effects (G or E). First, the raw counts were filtered, normalized, and transformed as described here previously. Each genotype was assigned to one of three genetic clusters by the maximum posterior probability of STRUCURE and DAPC analysis. In brief, we fit negative binomial models for each gene and run likelihood ratio tests (LRT) comparing the full model with reduced model by dropping the factor of interest. Subsequently, we conducted post hoc analysis on significantly DEGs of this omnibus test to test for G, E, and G×E effect of each of the three pairwise comparisons with appropriate contrast matrix by Wald test. Subsequently, we estimated log2 fold change (LFC) of each pairwise contrast separately for each factor (G or E) using the appropriate contrast. To partition the variance components for G, E, and G × E, we used the variancePartition package from Bioconductor (Hoffman and Schadt 2016) on voom-transformed data. Variance components were plotted as ternary plots using a customized R script. To identify DEG with consistent site effects among and between genetic clusters, we used LRT for fixed site effect (E) at each pairwise genetic cluster combination.

In order to estimate the plasticity of each genetic cluster due to transplantation, we implemented DAPC function using the adegenet (v2.0.1) package. Linear discriminant function was built by defining native genetic clusters at native sites as separate groups (inland genotypes at the inland site and coastal genotypes at the coastal site) on the total transcriptome space. DAPC uses higher-order PCA to maximize variation between predefined groups while minimizing the variance within groups. We measured the plasticity of a focal genotype as the absolute difference between the DAPC value (*z*-transformed at each genetic cluster level) at its transplant and native sites. We tested the effect of genetic clusters on gene expression plasticity by fitting a linear mixed model with genetic cluster as a fixed effect and genotypes as random effects. The mean plasticity of each genetic cluster was presented by the arrow length in fig 2*E*.

To determine whether a particular expression genetic architecture is predominant within a specific class of DEGs, we conducted an enrichment analysis involving distinct categories of eQTL (including *cis*-eQTL, *trans*-eQTL, *cis*-eQTLxdrought, and *trans*-eQTxdrought), as previously identified by Lovell et al. (2018). Employing Fisher's exact test, we examined the frequency of a specific eQTL category within a given class of DEGs and compared it with the frequency for the expressed background genes.

### Detection of Selection on Gene Expression

We implemented the *Q*_PC_ method of Josephs et al. (2019) to detect natural selection in structured populations using kin relationships determined from genomic data. This method is an extension of the *Q*_ST_–*F*_ST_ method where the additive genetic variance (*V*_A_) is estimated by the orthogonal PCs of the population kinship matrix. The method has been previously used to detect excessive expression divergence in maize (Blanc et al. 2021). The kinship matrix was constructed and mean-centered using the same set of 50,000 neutral SNPs that were used to construct genetic clusters. Matrix construction and standardization was performed following the method described in Josephs et al. (2019). PCs (1 through 5) that cumulatively explained the top 25% of variation in the conditional kinship matrix were chosen for *Q*_PC_ testing. Later, PCs which explained cumulatively ∼50% of the total variance of the kinship matrix were used as representative of the neutral evolution. We implemented the *Q*_PC_ test on the mean-centered normalized mean of across sites and the difference between sites of each expressed gene. This strategy relied on reaction norm perspective: mean expression is equivalent to studying the main effect of genetic clusters (hereby identified as “constitutive”), whereas the difference is equivalent to the interaction of genetic clusters with planting location (hereby identified as “plastic”). First, TagSeq libraries were normalized for their library size, were variance stabilizing transformed which uses the fitted dispersion–mean relationship in order to achieve homoscedasticity and then obtain the mean across and difference between sites for each accession. CI for a given PC for a given trait was estimated as described by Josephs et al. (2019). FDR-adjusted *q*-values were calculated using qvalue function in R with an FDR threshold of 0.1 (Storey and Tibshirani 2003; Storey et al. 2004). As mentioned earlier, we observed very low recombination for the heterochromatin regions of the genome compared with euchromatin regions which could alter the expected neutral evolution between these two regions. Therefore, we carried out *Q*_PC_ tests in two different approaches: 1) relaxed mode, where we applied *Q*_PC_ method on all expressed genes (18,773), and 2) constrained mode, only on selected genes residing in highly recombined regions (15,735). For the constrained mode, we also purged the SNPs which were located in nonrecombining regions (7,562 SNPs) and constructed the kinship matrix as described for relaxed mode.

### Ortholog Identification

Orthologs among *P. hallii* HAL2 and FIL2 ecotype genome references (HAL2 v2.1: https://phytozome-next.jgi.doe.gov/info/PhalliiHAL_v2_1; FIL2 v3.1: https://phytozome-next.jgi.doe.gov/info/Phallii_v3_1) were identified by OrthoFinder version 2.5.4 applying default parameters except selecting “Blast” as the sequence search algorithm. This program uses a heuristic analysis of pairwise sequence similarity to estimate the phylogenetic relationship between genes and infer putative orthologous clusters. We only considered one-to-one ortholog pairs for downstream analysis of sequence evolution to avoid potential complications from paralogous relationships. We also eliminated ortholog pairs with average percent identity below 50% or with >10% difference of protein length in order to exclude misalignment or erroneous orthologous relationship. In this way, we detected 19,965 one-to-one ortholog pairs in *P. hallii*. In order to obtain orthologous relationships among the broader *Panicum* lineage, we also considered the K and N subgenomes of allopolyploid *P. virgatum* as two additional genomes along with two genomes *of P. hallii* (HAL2 and FIL2) and implement the same OrthoFinder pipeline described above. In *Panicum*, we detected 8,395 one-to-one ortholog pairs considering all pairwise relationships. In order to infer the ancestry of inland and coastal populations, we further identified 7,419 one-to-one orthologs between inland and coastal references of *P. hallii*, two subgenomes of *P. virgatum*, and *S. viridis* by implementing the same orthology strategy described above.

### Phylogenetic Analysis and Ancestry Inference

To infer whether the Inland or Coastal genetic cluster is ancestral, we first aligned 7,419 one-to-one orthologs of *P. hallii*, *P. virgatum* (both N and K subgenomes), and *S. viridis* gene models for individual orthogroup by Clustal Omega (Sievers et al. 2011). Alignments were concatenated to construct the species tree using maximum likelihood–based algorithm, RAxML (version 8.2.12) (Stamatakis 2014), under PROTGAMMA model using species other than *P. hallii* as outgroups with 1,000 bootstrap replicates. In order to polarize the tree, we looked at 4,969 orthogroups which have at least 3 variant sites per alignment between inland and Coastal genetic clusters and for which ancestry can be inferred from outgroups. Subsequently, we estimated the frequency of coastal alleles as ancestral for orthogroups with 10,000 bootstrap replicates.

### Molecular Population Genetic Analyses

Our project generated high-quality resequencing data from 86 *P. hallii* genotypes for molecular population genetic analyses. To understand global genetic diversity among and between genetic clusters, we estimated genome-wide diversity statistics on 20 Kb window (window length = 20 Kb, step size = 20 Kb) for each pairwise comparison between genetic clusters (*F*_ST_ and *D*_XY_) or within each genetic cluster (π). We used VCFtools to estimate mean Weir and Cockerham's *F*_ST_. Nucleotide diversity (π) and the absolute measure of divergence between populations (*D*_XY_) were estimated for the same intervals by pixy tool (Korunes and Samuk 2021) using all detected sites (both high-quality SNP variants and quality filtered nonvariant sites). One-way ANOVA followed by Tukey's post hoc tests were performed to test for differences of these statistics between different pairwise contrasts (or between different genetic clusters for π). Later, we also estimated these statistics split by different gene features (CDS, promoter, intron, 5′-UTR, and 3′-UTR) along with complete gene models of one-to-one ortholog pairs which were detected as expressed in our study using HAL2 v2.1 annotation (https://phytozome-next.jgi.doe.gov/info/PhalliiHAL_v2_1).

We determined the pairwise divergence of nonsynonymous and synonymous sites and their ratio in the coding regions of 13,223 one-to-one ortholog genes in *P. hallii* which were detected as expressed in our study using the CODEML function of PAML tool (version 4.9; runmode −2, F3X4 codon frequency). Alignment sites with gaps were removed, and ortholog pairs shorter than 50 amino acids were purged. Low divergence at synonymous sites may inflate or overestimate the dN/dS ratio; therefore, ortholog pairs with dS < 0.01 were also excluded. Mann–Whitney two tests were performed to detect for the difference of mean dN/dS between CDE and NDE group. Partial correlation tests (Spearman's test) for constitutive divergence expression with substitution rate were performed by “pcor” function in R while controlling for mean expression across site. CDE (=1) and NDE (=0) genes were dummy coded to obtain divergence score.

In order to obtain more power to detect site-specific positive selection, we included two subgenomes of *P. virgatum*, and we used site models using CODEML function of PAML (F3X4 codon frequency). An unrooted species tree was provided, and dN and dS at each codon across all branches in the tree were estimated. We tested for sites evolving by positive selection (ω = dN/dS is >1) by comparing model M1a (nearly neutral) and model M2a (positive selection) using a LRT with twice the difference of the log-likelihood values of the M1a to M2a model χ^2^ distributed with two degrees of freedom. We used a significance threshold alpha = 0.05, and multiple testing correction was implemented by Bonferroni method.

## Supplementary Material

msad210_Supplementary_Data

## Data Availability

The TagSeq and resequencing data have been deposited in the National Center for Biotechnology Information (NCBI) short read archive (SRA). The TagSeq raw reads were deposited to SRA under BioProject ID PRJNA915463. The details of BioProjects, sample IDs, and metadata can be found in supplementary tables S1 and S14, Supplementary Material online. Codes for the analyses have been deposited in GitHub (https://github.com/BhaskaraGB/PHtrans).
