# Supplementary material for: Evolutionary Analyses of Gene Expression Divergence in Panicum hallii: Exploring Constitutive and Plastic Responses Using Reciprocal Transplants
Source: Mol Biol Evol. 2023 Sep 20;40(10):msad210. doi: 10.1093/molbev/msad210 (PMC10556983; doi:10.1093/molbev/msad210)
Supplement: msad210_Supplementary_Data [file msad210_supplementary_data.zip › Supplemental_Figures_Final Update.pdf]

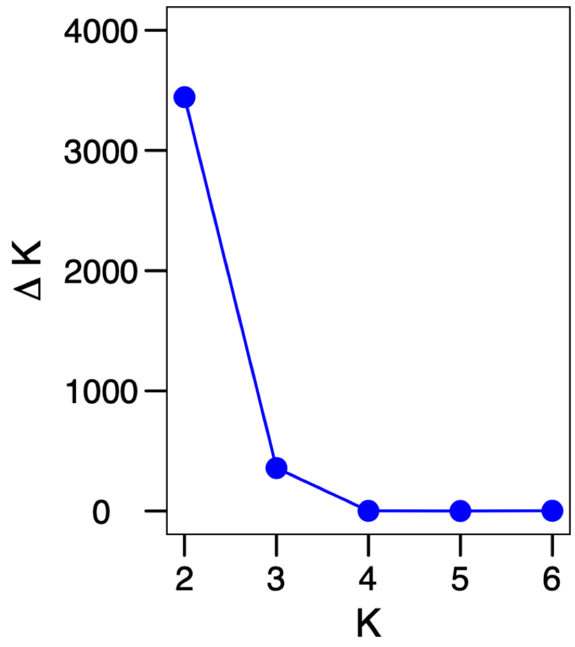

Figure S1. Graph of ΔK values to determine the most likely number of groups (K) for the STRUCTURE analysis for all Panicum hallii. The highest ΔK value for this analysis and corresponding K were chosen to represent the number of groups for downstream analyses.

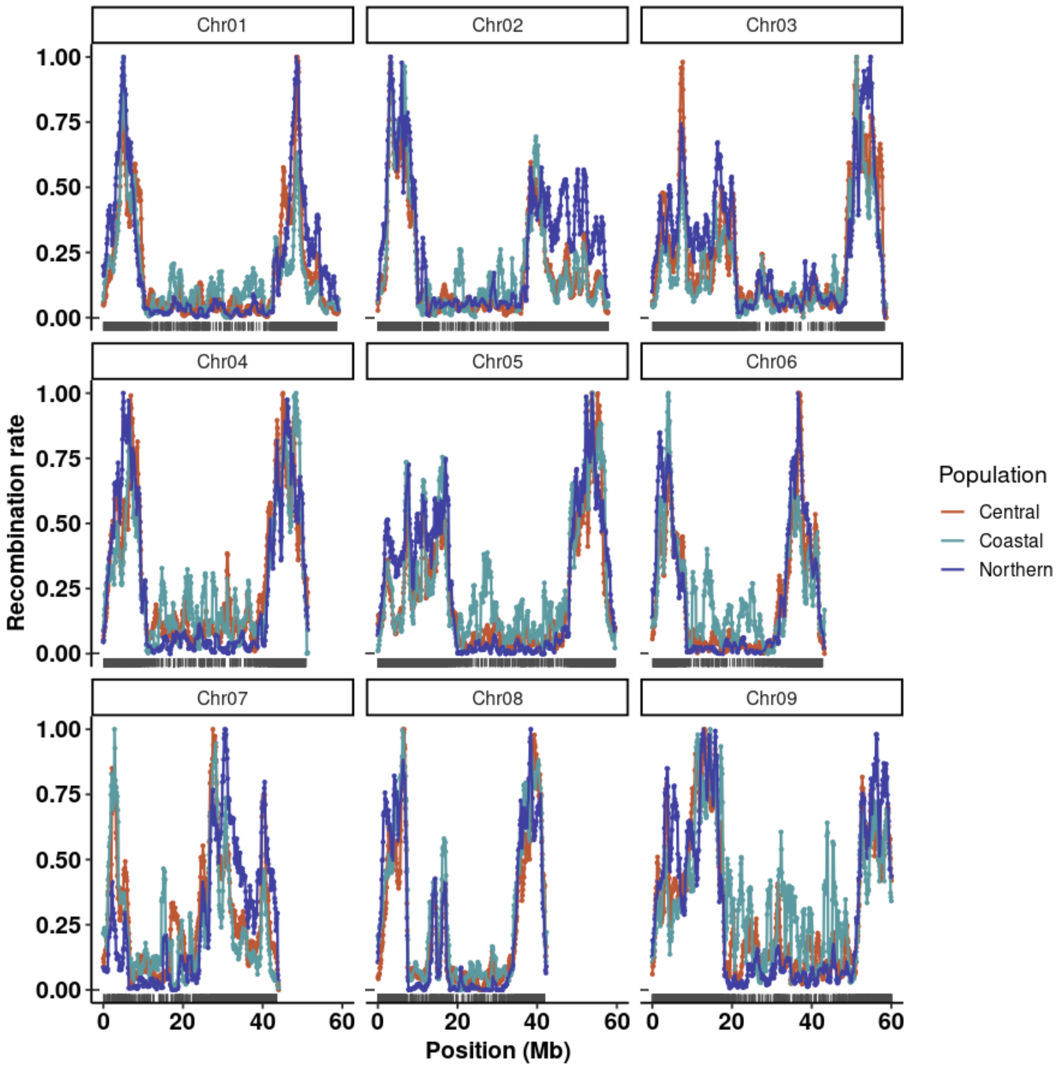

Figure S2. Recombination landscape of individual genetic cluster across chromosomes. Population-scaled recombination rate was plotted on y-axis and x-axis represent the physical position of the chromosome. The rug at the bottom of the figure represents gene density.

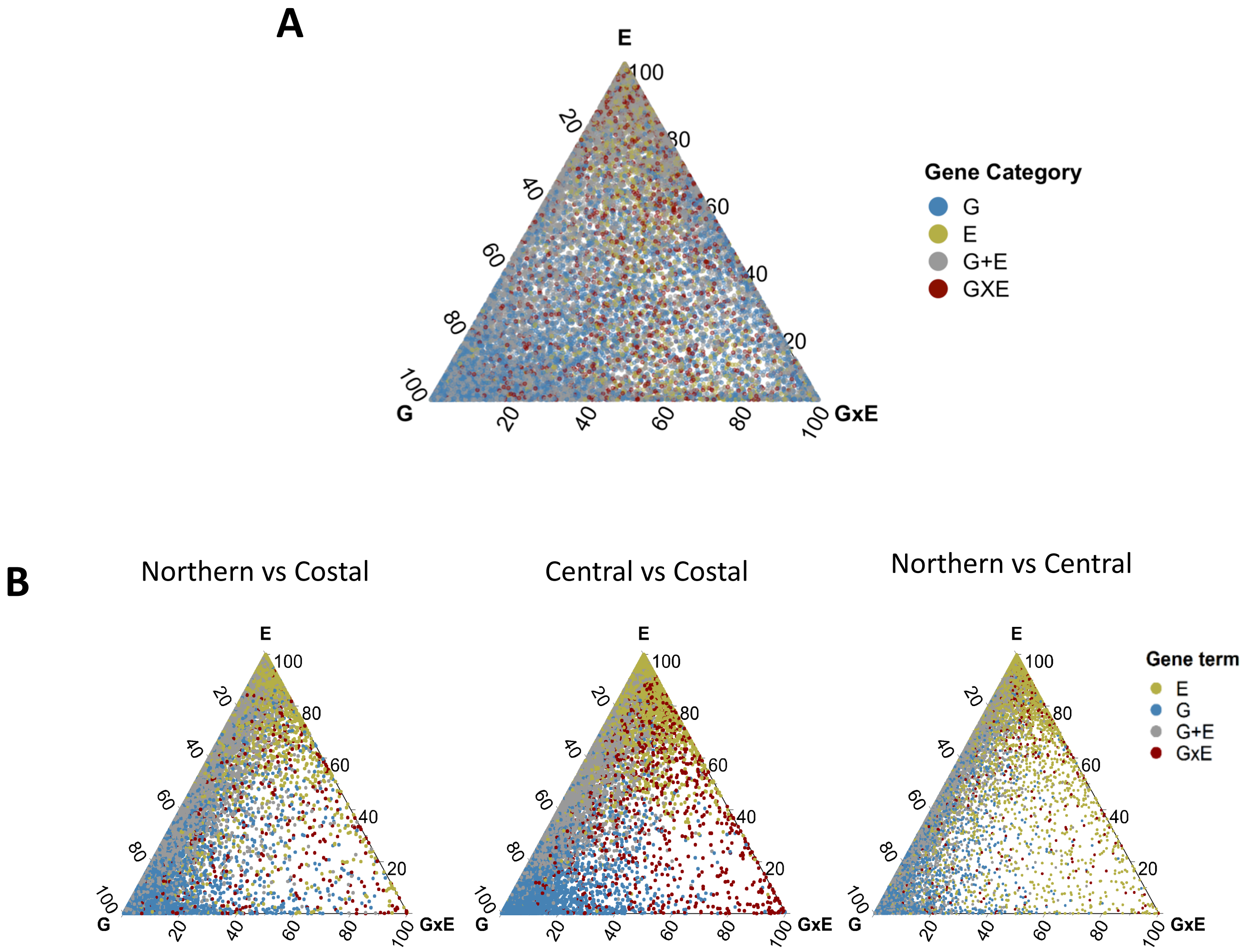

Figure S3. Ternary plots describing the percent of variance in gene expression explained by G, E, G+E and G×E, (A) Global analysis by omnibus test, B) Post-hoc analysis by pairwise genetic clusters comparison. We estimated percent variance explained by each model terms by fitting a linear mixed model. Genes were shown in colored points based on their different significant likelihood ratio tests (LRT) (G, E, G+E or G×E) (B) Genes that are differentially expressed (FDR-adjusted P-value ≤ 0.05) for the given contrast are plotted in the ternary diagram.

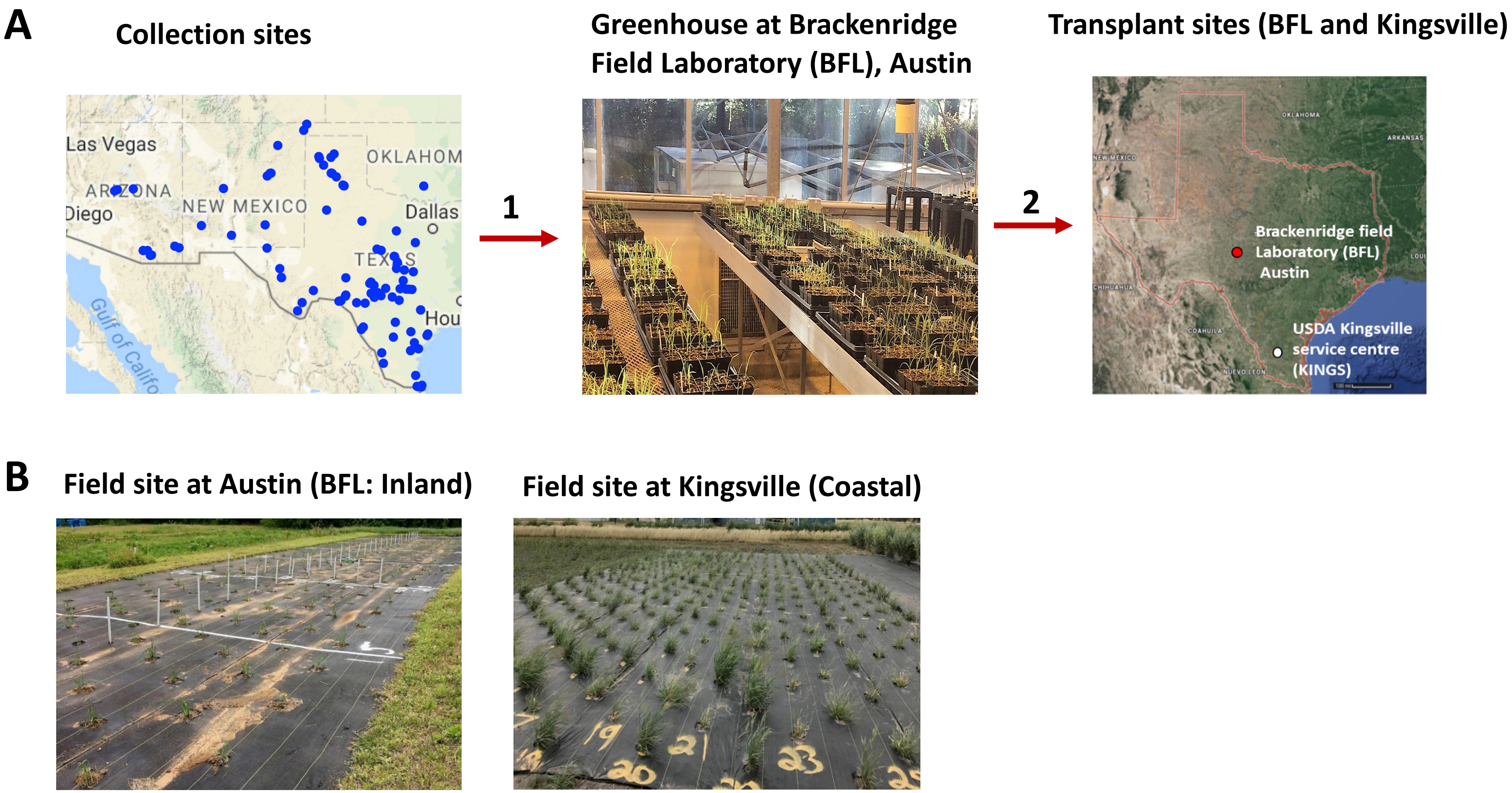

Figure S4. Figure outline explains experimental setup of reciprocal transplantation experiment (A) *P. hallii* accessions were collected across northwestern Texas westward into New Mexico and Arizona, and central to south Texas regions. Germination and seedling establishment was carried out in greenhouse at Brackenridge Field Laboratory (BFL), Austin (1). Young seedlings were then reciprocally planted at two field sites: one at BFL, at Austin representing an inland habitat and another at USDA service center, Kingsville, representing coastal habitat (2) (B) Photos of field sites with *P. hallii* planted at BFL and KINGS.

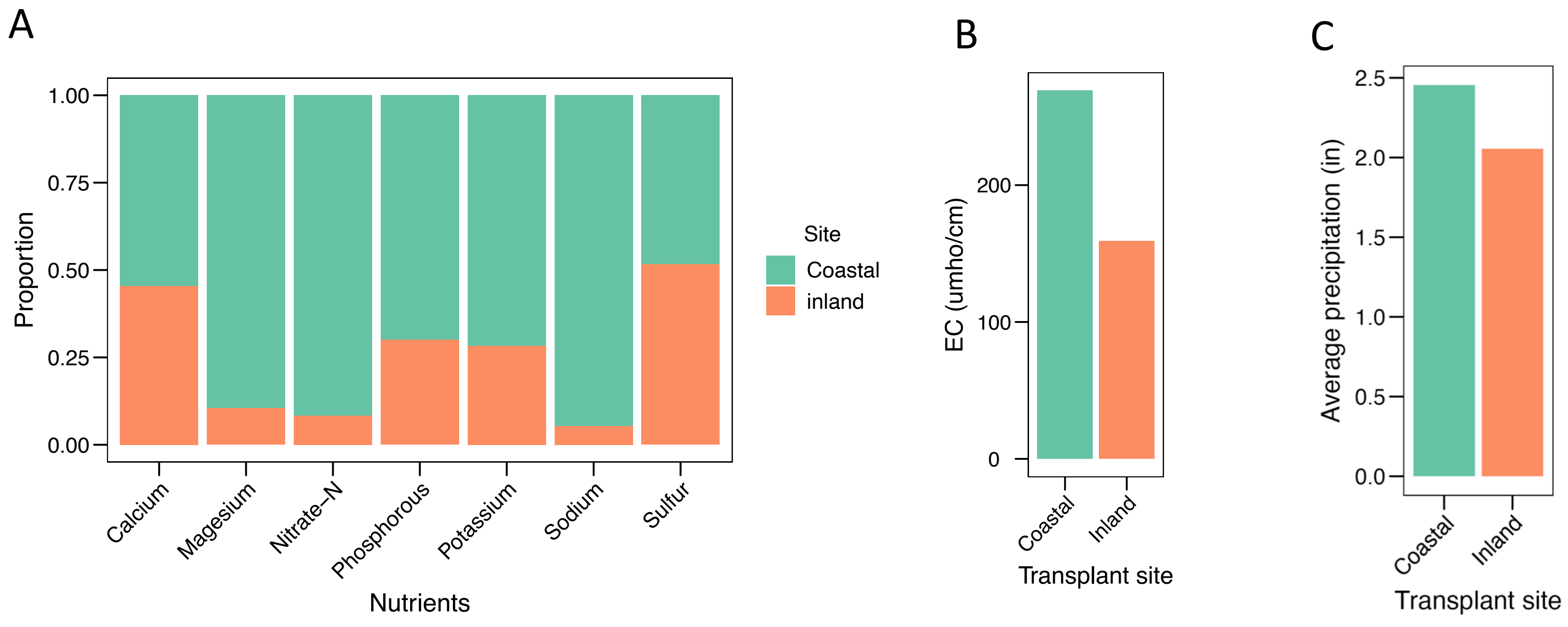

Figure S5. Soil composition and average precipitation of transplant field sites (A) Soil nutrients (Values are scaled to 1) (B) Soil electrical conductivity (EC) (C) Average precipitation (inch)
